# An invisible villain: high perceived stress, its associated factors, and possible consequences in a population-based survey in southern Brazil

**DOI:** 10.47626/2237-6089-2021-0228

**Published:** 2022-09-13

**Authors:** Lauro Miranda Demenech, Sara S. Fernandes, Renata Gomes Paulitsch, Samuel C. Dumith

**Affiliations:** 1 Programa de Pós-graduação em Ciências da Saúde Universidade Federal do Rio Grande Rio Grande RS Brazil Programa de Pós-graduação em Ciências da Saúde, Universidade Federal do Rio Grande (FURG), Rio Grande, RS, Brazil.

**Keywords:** Perceived stress, risk factors, consequences, etiologic fraction, epidemiology

## Abstract

**Introduction:**

Much of the evidence on the relationship between stress, lifestyle, and other physical and mental health outcomes comes from studies conducted in high-income countries. There is therefore a need for research among populations in low and middle-income settings.

**Objectives:**

To measure stress levels and identify factors associated with a high stress level and its consequences for health.

**Methods:**

This was a population-based cross-sectional study carried out in 2016 with adults aged 18 years or older in a municipality in southern Brazil. A two-stage sampling strategy based on census tracts was used. Stress levels were measured with the Perceived Stress Scale (PSS-14) and classified into quartiles. The impact of the highest stress levelon each outcome was assessed with etiologic fractions (EF).

**Results:**

The most stressed groups were: females (PR = 1.51, 95%CI 1.25-1.81), younger people (PR = 1.76, 95%CI 1.26-2.46), middle-aged individuals (PR = 1.60, 95%CI 1.17-2.19), those with lower schooling (PR = 1.56, 95%CI 1.20-2.02), the physically inactive (PR = 1.51, 95%CI 1.20-1.91), people who spent three or more hours watching television per day (PR = 1.29, 95%CI 1.12-1.50), and those with food insecurity (PR = 1.44, 95%CI 1.19-175). Possible consequences of high stress level were regular or poor self-perception of health (EF = 29.6%), poor or very poor sleep quality (EF = 17.3%), lower quality of life (EF = 45.6%), sadness (EF = 24.2%), and depressive symptoms (EF = 35.8%).

**Conclusions:**

Stress plays an important role in several domains of health. Both public policies that target reduction of inequalities and specific stress-management interventions can reduce stress levels in populations, thereby decreasing the burden of other negative physical and mental health outcomes related to stress.

## Introduction

Stress can be defined as the body’s response pattern to external demands, regardless of the nature of the causative agent, and the implications of stress are of considerable interest in health research.^1^ While responses to acute stress are adaptive, chronic stress can predispose individuals to a lower quality of life and increased health problems.^2^ Evidence indicates that stress can hinder development of a healthy lifestyle. People under stress are more likely to adopt harmful health behaviors, such as physical inactivity, smoking, and drinking alcoholic beverages.^3^ Stress seems to have a complex and bidirectional relationship with mental disorders, especially depression.^4^

Much of the available evidence on the relationship between stress, lifestyle, and other physical and mental health outcomes comes from studies conducted in high-income countries.^5 - 7^ There is therefore a need for research among populations in low and middle-income settings. There are also complex interrelations between individual (gender, education, occupation, income, behaviors) and contextual factors (structure, culture, and values of the region or country in which one lives) that predispose a person to be more stressed. Failure to account for these mechanisms may lead to incomplete interpretations of possible adverse outcomes resulting from stress.

High levels of perceived stress have been associated with poorer overall physical and mental health, in addition to increased risk of premature death.^6^ Investigating the association between psychological stress and health-related outcomes is of foremost relevance. Mapping how stress can be shaped according to individuals’ characteristics and to modifiable lifestyle behaviors, as well as its effects, can provide health professionals and key stakeholders with helpful insights and information for development of better health plans, policies, and practices.

Therefore, the objective of the present study was to identify the social, economic, demographic, and behavioral factors that are associated with perceived stress and to investigate the possible consequences of stress for the physical and mental health of the population of a municipality in southern Brazil.

## Materials and methods

### Study design

This cross-sectional study is part of a population-based study, titled “Health of the Population of Rio Grande” (Saúde da População Rio-Grandina]. The aim of this research was to evaluate the health of adults and the elderly in the city of Rio Grande in southern Brazil.

The project was approved by the Research Ethics Committee at the Universidade Federal do Rio Grande (protocol 20/2016; CAAE: 52939016.0.0000.5324).

### Sampling plan

The sample size estimate was calculated by considering the different outcomes evaluated in the research project. The parameters considered were as follows: significance level of 5%, power of 80%, prevalence of outcome of 10%, frequency of exposure between 20 and 60%, and prevalence ratio of 2.0. Then, 50% was added to the sample size estimate to account for the sampling design effect (because sampling units were households, resulting in a cluster effect by which members of the same household tend to have more similar characteristics and responses), 15% was added to account for possible confounders, and 10% was added to account for attrition and refusals. The final sample size consisted of 1,423 individuals.

The sampling process was conducted in two stages based on the 2010 Demographic Census Data.^8^ First, 70 of the 293 census tracts in the municipality (approximately 25%) were systematically selected. Considering that it was expected that there would be an average of two individuals aged 18 years or over per household,^8^ 711 households (1,423 ÷ 2) were selected, with a probability proportional to the size of the census tract. All individuals aged 18 years or older in each of the households selected were invited to take part in the study.

### Procedures

First, the study was publicized via interviews on local radio stations, publications in local newspapers, and television newscasts, and on a social media page on the internet. Second, preliminary visits were made to selected households by study supervisors, in order to verify household characteristics (whether it was a residential address and to determine number of residents eligible for the study), to inform residents about the study, and to schedule interviews. Data collection was conducted between April and July 2016 using a precoded and standardized questionnaire that had been tested previously and was administered by trained interviewers. People who agreed to participate signed a consent form. Data on sex and age were collected from those who did not agree to participate. Data were input twice by different supervisors using EpiData 3.1 software. More details regarding the sample size calculation, sampling plan, and fieldwork logistics can be found elsewhere.^9^

### Variables and instruments

The outcome was perceived stress, measured using the Perceived Stress Scale,^10^ which is a 14-item instrument that assesses the frequency with which an individual has experienced certain feelings and situations. This scale has been translated and validated for use in the Brazilian setting.^11^ A Likert scale was used, with response options ranging from 0 (never) to 4 (always) points. A total score ranging from 0 to 56 points is generated by summing the scores for all questions. The variable perceived stress was operationalized in quartiles based on the total score. The top quartile was considered the group with the highest stress levels (the most stressed individuals).

Independent variables included in the analyses were sex, age, skin color, marital status, living alone, schooling level, wealth index (generated through a principal component analysis with 11 items of home assets or house characteristics and then categorized into tertiles), smoking, excessive alcohol consumption (five or more drinks for men and four or more drinks for women on a single occasion in the previous 30 days^12^ ), physical activity during leisure time (some or none), hours per day watching television, food insecurity (defined as any score above zero on the Brazilian Scale of Food Insecurity^13^ ), and whether the respondent had visited a physician in the previous month.

The possible physical and psychological consequences of stress evaluated herein were back pain in the previous 12 months (self-reported, no/yes), obesity (self-reported body mass index ≥ 30 kg/m^2^ ), self-perception of health (excellent, very good or good/regular or poor), quality of sleep (very good, good or regular/poor or very poor), quality of life as measured by the World Health Organization instrument validated in Brazil (total score operationalized into quintiles),^14^ sadness as measured by the Andrews and Whitney face scale (defined as those who reported feeling unhappy or very unhappy),^15^ depressive symptoms (absent/present) as measured by the Patient Health Questionnaire (PHQ-9) validated in the Brazilian population,^16^ and self-reported medical diagnosis of hypertension, diabetes or cardiopathy (no/yes). It is worth noting that the term “possible consequences” refers to outcomes that are theoretically believed to have increased probability of occurrence when levels of stress are high, but causality cannot be determined within the constraints of the design of this study.

### Data analysis

**Figure 1:**
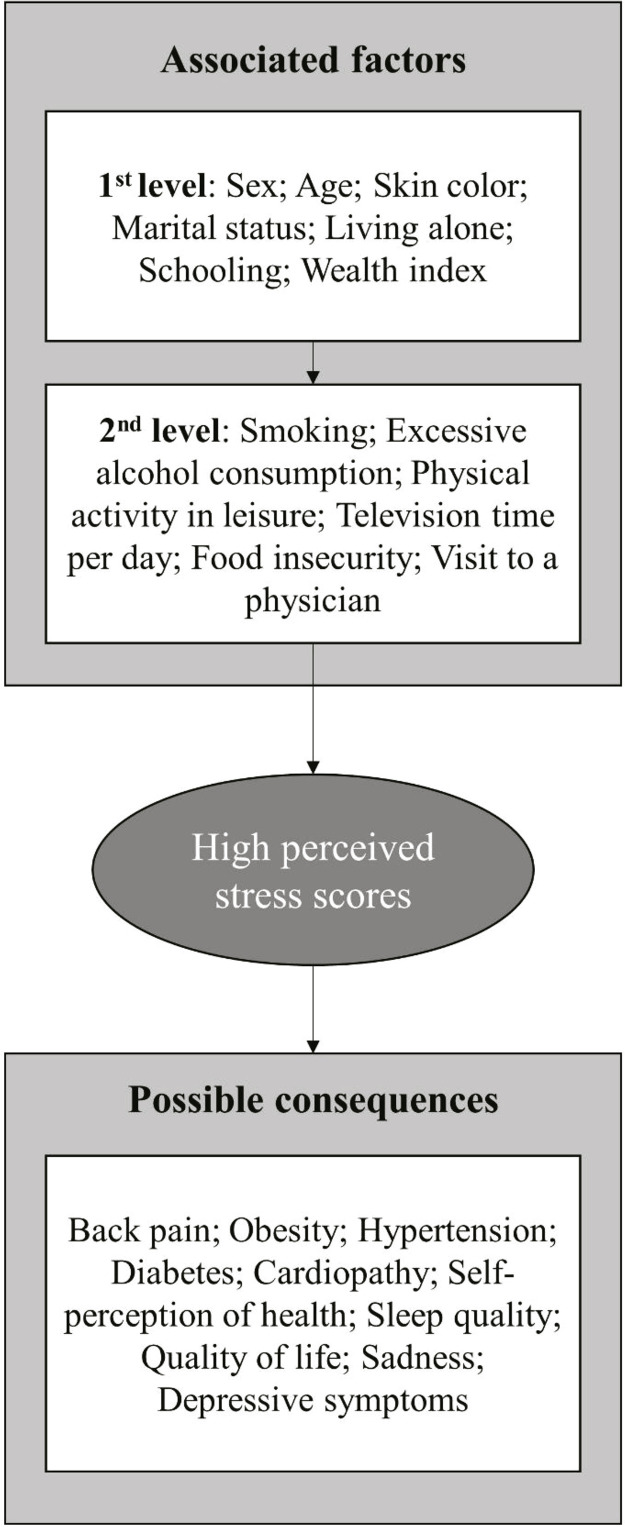
Conceptual model of analysis of associated factors and possible consequences of high stress levels. Rio Grande, Brazil, 2016 (N = 1,295).

The statistical analyses were conducted in Stata 15.1. First, a descriptive analysis of sample characteristics was performed. After this step, bivariate analyses were performed to calculate the proportions of highly stressed individuals (top quartile) according to each of the independent variables. Then, a multivariate analysis was carried out with Poisson regression with robust adjustment of variance to identify the factors associated with the highest stress level or the consequences of the highest stress level.^17^ The conceptual model for these analyses is illustrated in Figure 1 .

For the multivariate analysis, a hierarchical model was constructed on two levels by the backward method ( Figure 1 ).^18^ Variables with a p-value < 0.20 were retained in the final model. The remaining variables were used for adjustment purposes to verify the effects of the highest stress level on the different outcomes (Model 1). The analysis of the consequences of stress was also adjusted for all other outcomes (Model 2) to verify the effect of stress on each outcome independently of the other outcomes. The results of crude and multivariate analyses are presented as prevalence ratios (PR) and 95% confidence intervals (95%CI).

Finally, we calculated the etiologic fraction (EF) of the stress on the physical and psychological outcomes using the formula . The EF is interpreted as the proportion of the outcome that might be reduced if the group with the highest stress level were omitted from the population. All analyses were performed considering the sample design effect and a significance level of 5% for two-tailed tests.

### Data availability statement

We declare that the data used in this manuscript is available upon request from the corresponding author.

## Results

The sample comprised 1,295 individuals, which corresponds to a response rate of 91.0%. Among non-respondents (9%), 6.9% were refusals and 2.1% were losses. The proportion of males was significantly higher among non-respondents than among participants (p < 0.001), with no significant difference in age (p = 0.64). Majorities of the sample were female (56.6%), had white skin color (82.9%), and were not living alone (90.4%). Almost half of the sample (41.8%) had eight years or less of schooling, 39.3% were aged between 18 and 39 years, 11.7% reported excessive alcohol consumption, 17.9% were current smokers, 31.8% watched television for three or more hours per day, and 33.3% engaged in some physical activity. Approximately one-third of the sample reported food insecurity and had visited a doctor in the previous month ( Table 1 ). The mean perceived stress score was 23.6 points (standard deviation [SD] = 7.4), and scores ranged from 3 to 50 points. The cutoff for the quartile with the highest stress level was ≥ 29 points.

**Table 1:** Description of the characteristics of the sample of adults aged 18 years or older from Rio Grande, Brazil, 2016 (N = 1,295).

Variable	n	%
Sex (N = 1,295)		
Male	562	43.4
Female	733	56.6
Age (years) (N = 1,295)		
18 to 39	508	39.3
40 to 59	477	36.8
≥ 60	310	23.9
Skin color (N = 1,293)		
White	1,072	82.9
Black and others	221	17.1
Marital status (N = 1,295)		
Single	600	46.3
Not single	695	53.7
Living alone (N = 1,294)		
No	1,170	90.4
Yes	124	9.6
Schooling (years) (N = 1,293)		
0 to 8	541	41.8
9 to 11	397	30.7
≥ 12	355	27.5
Wealth index (tertiles) (N = 1,294)		
1st (poorest)	444	34.3
2nd	417	32.2
3rd (richest)	433	33.5
Smoking (N = 1,295)		
No	1,063	82.1
Yes	232	17.9
Excessive alcohol consumption (N = 1,292)		
No	1,140	88.2
Yes	152	11.8
Physical activity in leisure (N = 1,294)		
No	862	66.6
Yes	432	33.4
Television time (hours per day) (N = 1,279)		
< 3	872	68.2
≥ 3	407	31.8
Food insecurity (N = 1,295)		
No	841	64.9
Yes	454	35.1
Visit to physician (last month) (N = 1,295)		
No	879	67.9
Yes	416	32.1

% = prevalence; N = total number of observations per category; n = absolute frequency.

Table 2 shows the distribution of individuals with the highest stress level, grouped by the independent variables. Individuals with food insecurity had the greatest proportion of highly stressed individuals (35%), whereas individuals who were physically active had the lowest proportion of highly stressed individuals (16%). In the crude analyses, the variables age, marital status, schooling, wealth index, physical activity, time watching television, food insecurity, and having visited a doctor were significantly associated with the highest level of stress. In the adjusted analysis, the following variables remained significant: female sex (PR = 1.51, 95%CI 1.25-1.81), age between 18 and 39 years old (PR = 1.76, 95%CI 1.26-2.46) or between 40 to 59 years old (PR = 1.60, 95%CI 1.17-2.19), schooling less than or equal to eight years (PR = 1.56, 95%CI 1.20-2.02), physical inactivity (PR = 1.51, 95%CI 1.20-1.91), watching television for three hours or more per day (PR = 1.29, 95%CI 1.12-1.50), and food insecurity (PR = 1.44, 95%CI 1.19-175).

**Table 2:** Crude and adjusted prevalence ratios for associations between the highest stress level and independent variables. Multivariate analysis conducted with two hierarchical levels, through Poisson regression with robust adjust for variance, accounting for design effect. Sample of adults aged 18 years or older in Rio Grande, Brazil, 2016 (N = 1,295).

Level/variable	Prevalence* (%)	Crude analysis PR (95%CI)	Adjusted analysis PR (95%CI)
First level			
Sex			
Male	19.4	1.00	1.00
Female	28.8	1.48 (1.23; 1.80)	1.51 (1.25; 1.81)
Age (years)			
18 to 39	28.5	1.70 (1.30; 2.24)	1.76 (1.26; 2.46)
40 to 59	25.8	1.54 (1.13; 2.10)	1.60 (1.17; 2.19)
≥ 60	16.8	1.00	1.00
Skin color			
White	23.7	1.00	1.00
Black and others	29.4	1.24 (1.00; 1.55)	1.13 (0.90; 1.42)
Marital status			
Single	29.3	1.42 (1.13; 1.78)	1.23 (0.94; 1.61)
Not single	20.7	1.00	1.00
Living alone			
No	25.5	1.00	1.00
Yes	17.7	0.70 (0.47; 1.04)	0.72 (0.47; 1.10)
Schooling (years)			
0 to 8	27.9	1.42 (1.11; 1.80)	1.56 (1.20; 2.02)
9 to 11	24.7	1.25 (0.96; 1.63)	1.27 (0.98; 1.63)
≥ 12	19.7	1.00	1.00
Wealth index (tertiles)			
1st (poorest)	30.2	1.52 (1.18; 1.96)	1.30 (0.99; 1.71)
2nd	24.0	1.21 (0.92; 1.59)	1.06 (0.81; 1.39)
3rd (richest)	19.9	1.00	1.00
Second level			
Smoking			
No	23.8	1.00	1.00
Yes	28.9	1.21 (0.96; 1.53)	0.99 (0.77; 1.26)
Excessive alcohol consumption			
No	25.4	1.00	1.00
Yes	20.4	0.81 (0.59; 1.09)	0.91 (0.66; 1.25)
Physical activity in leisure			
No	29.0	1.82 (1.42; 2.33)	1.51 (1.20; 1.91)
Yes	16.0	1.00	1.00
Television time (hours per day)			
< 3	22.4	1.00	1.00
≥ 3	30.2	1.35 (1.15; 1.59)	1.29 (1.12; 1.50)
Food insecurity			
No	19.1	1.00	1.00
Yes	35.0	1.83 (1.51; 2.22)	1.44 (1.19; 1.75)
Visit to physician (last month)			
No	22.8	1.00	1.00
Yes	28.9	1.27 (1.04; 1.55)	1.14 (0.91; 1.44)

95%CI = 95% confidence interval; PR = prevalence ratio.

* Prevalence of the highest perceived stress quartile by category.

Table 3 shows the prevalence of physical and psychological outcomes and their associations with the highest stress level. The most frequent outcomes were regular or poor self-rated health (33.7%), hypertension (28.1%), and obesity (23.7%). In the crude analysis, the highest stress level was significantly associated with back pain, cardiopathy, regular or poor self-rated health, poor or very poor quality of sleep, lower quality of life, sadness, and depressive symptoms. In the adjusted analysis, controlling for possible confounders (Model 1), the highest stress level was still significantly associated with these variables in addition to being associated with hypertension. In Model 2 (also adjusting for other outcomes), the highest stress level remained associated with regular or poor self-perception of health (PR = 1.53, 95%CI 1.29-1.81), poor or very poor quality of sleep (PR = 1.62, 95%CI 1.09-2.40), lower quality of life (PR = 2.70, 95%CI 2.05-3.05), sadness (PR = 2.27, 95%CI 1.43-3.57), and depressive symptoms (PR = 3.02, 95%CI 1.95-4.68). The highest stress level stress levels showed a protective effect against obesity (PR = 0.75, 95%CI 0.58-0.97).

**Table 3:** Crude and adjusted analysis of possible consequences of high levels of stress. Sample of adults aged 18 years or older in Rio Grande, Brazil, 2016 (N = 1,295).

Outcomes	Prevalence* (%)	Crude analysis PR (95%CI)	Adjusted model 1^†^ PR (95%CI)	Adjusted model 2^‡^ PR (95%CI)	EF (%)
Back pain	20.7	2.06 (1.71; 2.47)	1.91 (1.55; 2.34)	1.20 (0.93; 1.56)	18.0
Obesity	23.7	1.11 (0.91; 1.35)	1.01 (0.81; 1.27)	0.75 (0.58; 0.97)	2.5
Hypertension	28.1	1.15 (0.96; 1.39)	1.26 (1.05; 1.51)	1.08 (0.88; 1.32)	4.0
Diabetes	7.0	1.30 (0.84; 2.00)	1.54 (0.98; 2.43)	1.06 (0.64; 1.75)	2.1
Cardiopathy	10.2	1.47 (1.01; 2.14)	1.69 (1.18; 2.42)	1.17 (0.85; 1.62)	4.6
Regular or poor self-perception of health	33.7	2.25 (2.00; 2.53)	2.08 (1.84; 2.34)	1.53 (1.29; 1.81)	29.6
Poor or very poor sleep quality	10.7	2.96 (2.21; 3.96)	2.76 (2.08; 3.66)	1.62 (1.09; 2.40)	17.3
Lowest quintile of quality of life	19.9	5.22 (4.16; 6.56)	4.53 (3.59; 5.71)	2.70 (2.05; 3.05)	45.6
Sadness	9.0	4.54 (3.18; 6.48)	3.67 (2.51; 5.37)	2.27 (1.43; 3.57)	24.2
Depressive symptoms	11.2	5.97 (4.18; 8.53)	5.04 (3.51; 7.22)	3.02 (1.95; 4.68)	35.8

95%CI = 95% confidence interval; PR = prevalence ratio.

* Prevalence of outcome.

^†^ Adjusted for sex, age, marital status, living alone, schooling, wealth index, physical activity in leisure time, television time per day, and food insecurity.

^‡^ Adjusted for the same variables in model 1 and all outcomes adjusted for each other; EF = etiologic fraction.

Regarding the EF, the highest stress level made a substantial contribution to most outcomes ( Table 3 ). The EF was 45.6% for lower quality of life, 35.8% for depressive symptoms, 29.6% for regular or poor self-rated health, 24.2% for sadness, and 17.3% for poor or very poor quality of sleep. The results showed that stress made a low, but still significant, contribution to occurrence of obesity (EF = 2.5%).

## Discussion

### Main finding of this study

This study evaluated perceived stress levels of the population of a municipality in southern Brazil and attempted to identify the possible risk factors for and the consequences of high levels of stress. The mean perceived stress score in this sample was 23.6 (SD = 7.4). It was shown that female, younger, and less educated individuals had a higher probability of being more stressed. Participants who were physically inactive, watched more television, and reported food insecurity had a higher probability of being more stressed.

One of the possible consequences of high levels of stress was self-rated regular or poor health. In addition to a modest association (PR = 1.53), stress explained 29.6% of the variation in this outcome. An unexpected result was that participants with the highest stress level had a lower probability of being obese, although stress levels explained a low proportion of the variance in this outcome (only 2.5%). One of the main consequences of high levels of stress in this study was a reduction in quality of life. The most stressed participants had a 170% greater probability of having a lower quality of life, and stress alone accounted for 45.6% of the variance in this outcome. In this study, the highest stress level was significantly associated with and explained 17.3% of the variance in poor or very poor sleep quality. The most stressed individuals were two and three times more likely to present symptoms of sadness and depression, respectively, than their less stressed counterparts. In addition, the highest stress level explained a high proportion of the variance in these outcomes (24.2% for sadness and 35.8% for depression).

### What is already known on this topic?

The stress scores reported in studies conducted in low and middle-income countries, such as Jordan (17.7)^19^ and India (19.3)^20^ were lower than that those found in this study. However, the scores reported in high-income countries, such as Italy (15.2), Germany (14.9), France (15.0), and Poland (17.6) were higher than that found in our study.^21^ In addition, the mean score for perceived stress in our investigation was similar to the score reported in a study conducted in Greece (25.4),^21^ but it should be noted that Greece was about to enter into a profound social and economic crisis when that study was conducted. Thus, it is plausible that population stress levels are closely related to the degree of social and economic development of the community, possibly due to the direct and indirect benefits of these resources on the general quality of life. In low and middle-income settings, income inequality, unequal distribution of job opportunities, and low-quality working conditions can erode the social cohesion that allows people to live and work together. This process may decrease social resources, trust, and civic participation, and increase crime and deterioration of public structures and institutions, increasing overall levels of stress in populations.

There is evidence that women report being more stressed than men,^22^ possibly due to hormonal influences and social issues,^23^ such as the devaluation of their work, the need for more working hours, and the objectification of their bodies.^24^ Studies indicate that older people have lower levels of anxiety, depression and stress, as well as higher levels of happiness, satisfaction, and well-being,^25 , 26^ which can be explained by an increase in wisdom and an increased ability to deal with daily life stressors.^27^ Finally, individuals with less education may have greater difficulty finding optimal occupations and attaining higher socioeconomic status, which may expose them to greater and more persistent psychosocial stressors.^28^

Physical activity has a bidirectional relationship with stress, since physically active individuals tend to be less stressed and, consequently, are more likely to remain active.^29^ Individuals who spend more time in front of television tend to have higher levels of sedentary behavior (i.e., sitting and/or lying down),^30^ which has also been strongly associated with high levels of stress.^31^ Respondents with food insecurity may experience higher levels of prolonged and toxic stress, as they lack basic resources for survival and citizenship.^32^ Furthermore, food insecurity may result in insufficient intake of nutrients, generating physiological sequelae that may predispose individuals to psychological suffering.^32 , 33^

With respect to the finding about obesity, the initial hypothesis was that individuals with high levels of stress would be more prone to obesity, since stress plays a role in its development and maintenance.^34^ Notwithstanding, the results found in this study may have occurred due to two phenomena. First, individuals who eat for comfort seem to achieve lower levels of perceived stress, which could result in people with higher BMI having lower perceived stress scores.^35^ Second, the results may be due to a negative confounding effect, because obese people tend to have a worse perception of health,^36^ are sadder and more depressed,^37^ and have worse quality of sleep^38^ and quality of life.^39^ It is therefore plausible that when we control for these variables in multivariate analysis, obese people can, in fact, be less stressed.

The association between stress level and poorer self-perceived health corroborates the literature that emphasizes that self-perceived health can be referred to as a health indicator.^40 , 41^ Regarding quality of life, both acute and chronic stress have effects that compromise health, which can affect people’s quality of life.^42^ Although low quality of life is not considered a morbidity, it is associated with a wide range of physical and mental health outcomes with corresponding implications for public health.^43^

It is important to state that sleep and stress can have a bidirectional relationship. Poor sleep quality can cause impairments such as chronic stress and multimorbidities,^44^ which, in turn, can increase sleep-related problems, increasing stress. Concerning mental health, stress increases the risk of developing physical and mental disorders^45^ and is strongly associated with depression^4 , 46 , 47^ and suicidal thoughts.^48^ There is a biological mechanism for these effects, since stress causes neurochemical, immunological, and autonomic changes related to emotional and cognitive regulation, which may lead to manifestations of depressive symptoms.^49^

### What does this study add?

It should be highlighted that this is the first study of the risk factors for and consequences of perceived stress to be conducted in a representative sample of a Brazilian municipality. Etiologic factor estimates of the possible consequences associated with high levels of stress can also be especially interesting because they enable us to forecast the proportion of each outcome that would be reduced if we were able to eliminate high levels of stress.

This sample had a high mean perceived stress score, especially when compared to samples from high-income countries. Individuals who were female, younger, less educated, physically inactive, and subject to food insecurity, and people who watched more television per day had a higher probability of being more stressed. Consequences related to the highest stress level were regular or poor self-perceived health, poor or very poor sleep quality, lower quality of life, sadness, and depression. Stress alone explained a large proportion of the variability in these outcomes. An unexpected result was that the highest stress level was associated with a lower probability of being obese, even though this association was weak and poorly explained by stress.

This article sought to present the importance that stress plays throughout several domains of health and relate it to a wide range of individual characteristics and consequences. Results of this investigation can have at least three implications: First, strengthening public policies that promote gender equality, education and occupation opportunities for younger individuals, and access to healthy food, physical activities, and diverse leisure options may reduce stress levels in the population. These may be interpreted as broad recommendations, but without these basic actions, targeted interventions are likely to be less effective (or ineffective). Second, stress seems to play an important role in the development of several negative health outcomes. It can therefore be used as a proxy to screen for psychological and physical comorbidities by health professionals, considering that more stressed people were more likely to report poor health, poor quality of sleep, lower quality of life, and sadness and depression. Third, specific interventions targeting reductions in stress (at the individual and collective levels) can reduce the burden of physical and psychological suffering, considering that stress alone contributed to a significant proportion of the abovementioned consequences. Including psychologists in the family health strategy could facilitate community access to mental health assessment, prevention, and treatment, improving people’s overall quality of life.

### Limitations of this study

The findings of this investigation should be interpreted in light of its limitations. First, all variables were measured through self-report, which might produce less precise results. However, most large-scale epidemiological studies collect data using self-report measures, enabling us to compare our results with existing findings. Second, work-related characteristics were not assessed in this investigation, and considering its possible impacts on stress, this should be considered as a limitation. Future research conducted within the same (or a similar) context should address this topic in the investigation. Work conditions may influence key factors significantly associated with stress identified in this study. Job opportunities can be unequally distributed according to gender, age, and educational level, especially in low and middle-income countries^28^ such as Brazil. It can therefore result in better (or worse) material conditions (access to household assets and availability of healthy food), and in higher (or lower) opportunities to engage in physical activities and in leisure activities (other than watching television). Finally, the data were collected in 2016. Despite possible concerns regarding timeliness, this study is still relevant because, apart from shedding light on an important issue, it registers stress levels in a population in the pre covid-19 pandemic setting, allowing future comparisons of scenarios.
